# Histobulin as a complementary but essential therapeutic for Intravenous Immune Globulin Therapy of Pfeiffer‐Weber‐Christian disease with multiple allergic diseases and its effects on allergic disease: A case report

**DOI:** 10.1002/ccr3.3681

**Published:** 2020-12-29

**Authors:** Geunwoong Noh

**Affiliations:** ^1^ Allergy and Clinical Immunology Center Cheju Halla General Hospital Jeju‐si Korea

**Keywords:** atopic dermatitis, histobulin, intravenous immune globulin, multiple food allergies, Pfeiffer‐Weber‐Christian disease

## Abstract

Histobulin is complementary to IVIG therapy but is an essential therapeutic for PWCD. Histoublin is recommended not only in atopic dermatitis and multiple food allergies but also in patients with multiple allergic diseases.

## Graphical abstract

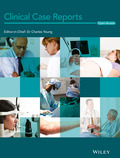

## 1 INTRODUCTION

A steroid‐ and cyclosporine A‐resistant case of Pfeiffer‐Weber‐Christian disease (PWCD) was remitted completely with sequential treatment with Histobulin and IVIG. The multiple food allergies and the atopic dermatitis of this patient with PWCD were also improved with Histobulin treatment. The upper respiratory infections were improved by Histobulin therapy also.

Pfeiffer‐Weber‐Christian disease (PWCD) is a rare relapsing nonsuppurative panniculitis that was described progressively by Pfeiffer,^1^ Weber,^2^ and Christian.^3^ IVIG shows immediate and dramatic effects in steroid‐ and cyclosporine A‐resistant PWCD as reported previously.

HistobulinTM (Green Cross PD, Korea) is a histamine‐fixed immunoglobulin preparation comprised of 0.15μg of histamine dihydrochloride and 12 mg of IgG.^4^ Histobulin is effective in allergic rhinitis and chronic urticaria.^5^ Recently, Histobulin was reported to be effective in atopic dermatitis.^6^ There are no previous reports that Histobulin is effective against PWCD. In this case, Histobulin was complementary but absolutely necessary for the remission of PWCD. Moreover, Histobulin therapy in this case had many interesting effects on the allergic diseases. The clinical effects of Histobulin on PWCD and allergic disease are described in this report.

## 2 CASE REPORT

A 35‐year‐old female patient visited the Department of Allergy and Clinical Immunology, Cheju Halla General Hospital (Jeju, Jeju Special Self‐Governing Province, Korea) because of allergic rhinitis and atopic dermatitis with a breast mass. Although she visited the clinic due to allergic disease, her main problem was the breast mass with excruciating pain that was suspected to be lymphoma needing chemotherapy. She had suffered from allergic rhinitis and atopic dermatitis for more than 20 years. She showed rhinorrhea, sneezing, tearing, and eczematous lesions on her entire body. Also, generalized urticaria and itching developed after eating crab, shrimp, and mackerel.

### 2.1 Histobulin therapy in multiple allergic diseases: Atopic dermatitis, multiple food allergy and allergic rhinitis: ‐ The 1st Histobulin therapy for the treatment of allergic diseases

Her atopic dermatitis fulfilled the Hanifin and Rajka criteria.^7^ Her clinical severity score for atopic dermatitis was evaluated before and after treatment using SCORad.^8^ The total score was 103 points. She received basic allergy laboratory tests such as complete blood counts with differential counts, serum eosinophil cationic protein, and serum total IgE level.

The specific IgE levels for 41 allergens were tested using a multiple allergosorbent test (MAST, Green Cross PD, Korea) including Dermatophagoides pteronyssinus (Dp), Dermatophagoides farina (Df), cat, dog, egg white, milk, soybean, crab, shrimp, peach, mackerel, rye pollen, house dust mites, cockroach, Cladosporium herbarum, Aspergillus fumigatus, Alternaria alternata, birch‐alder mix, white oak, short ragweed, mugwort, Japanese hop, hazelnut, sweet vernal grass, Bermuda grass, orchard grass, timothy grass, reed, Penicillium notatum, sycamore, sallow willow, poplar mix, ash mix, pine, Japanese cedar, acacia, oxeye daisy, dandelion, Russian thistle, goldenrod, and pigweed. The test results showed the levels of specific IgE for each allergen, and a normal negative range is 0.000‐0.349 IU/mL.

A skin prick test was also performed for 53 allergens. The allergens tested by the skin prick test were Alternaria alternaria, Aspergillus fumigatus, Aspergillus niger, Candida albicans, Cladosporium, Penicillium chrysogenum, German cockroach, Dp, Df, dog, cat, gray elder/silver birch, grass mix, mugwort, short ragweed, black willow pollen, orchard grass, Bermuda grass, timothy, English plantain, English rye grass, Holm oak, Japanese cedar, cotton flock, milk mix, egg mix, chicken, beef, pork, cod, oyster, salmon, prawn, mackerel, tuna, almond, peanut, bean, carrot, cabbage, walnut, maize, peach, tomato, black pepper, spinach, wheat flour, rabbit, kapok, hop, F acacia, pine, and poplar. Skin prick tests were conducted on the patient's back. Histamine hydrochloride 1 mg/mL was used as a positive control, and physiologic saline was used as a negative control. The results were measured as the wheal size. Reactions were read after 15 min and described as negative (0, no reaction), 1+ (reaction greater than control reaction but smaller than half the size of histamine), 2+ (equal to or more than half the size of histamine), 3+ (equal to or more than the size of histamine), and 4+ (equal to or more than twice the size of histamine). The minimum size of a positive reaction is 3 mm.

Her initial SCORad score was 89.5 points (Table 1). In the initial laboratory tests, the results of complete blood counts with differential counts and serum eosinophil cationic protein were within the normal limits. Her serum total IgE was very high at 2500 IU/mL. She showed she was diagnosed previously in other hospital with a food allergy to crab, shrimp, and mackerel, and she showed generalized urticaria and itching after intake of crab, shrimp, and mackerel. Her final diagnosis concerning her allergies was multiple allergic diseases, including allergic rhinitis, atopic dermatitis, and multiple food allergies. For the treatment of her allergic rhinitis, Histobulin therapy was begun. Before Histobulin therapy, her serum IgA level was checked as normal at 125.1 mg/dL to exclude a selective IgE deficiency to avoid an anaphylactic reaction.

**Table 1:** The significant laboratory results

Laboratory results		Before	After 1st cycle	After 2nd cycle
SCORad index (Points)		89.5	22.7	0
Total IgE (IU/ml)		2500	1737	2277
Open food challenges	Crab	Positive		Negative
	Shrimp	Positive		Negative
	Mackerel	Positive		Negative
Specific IgE (IU/ml)	Dp	31.65		100
	Df	8.16		3.29
	Cat	2.09		1.80
	Dog	100		100
	Crab	23.83		11.94
	Mackerel	16.36		0
	Short ragweed	0		1.44
	Orchard grass	0		0.77
	Japanese cedar	0		1.64
Skin prick test	Dp	3x2		6x6
	Df	3x3		6x5
	Dog	0x0		6x5
	Cat	0x0		4x3
	Short ragweed	0x0		8x7
	Japanese cedar	0x0		7x6
	Histamine	5x4		6x5
	Negative control	0x0		0x0

The patient received 2 mL of Histobulin by subcutaneous injection in the deltoid area of the upper arm every week 35 times in the 1st cycle. After the 1st cycle of Histobulin therapy, her SCORad index was dramatically improved to 22.7 points (Table 1). In the follow‐up laboratory test, the results of her complete blood counts with differential counts and serum eosinophil cationic protein were normal. Her serum total IgE levels were decreased to 1737 IU/mL. All symptoms and signs of allergic rhinitis were quite improved.

At this point, her diagnosis of PWCD was confirmed and during IVIG therapy for PWCD, the Histobulin therapy was stopped for 3 months. Then, the second cycle of 2nd Histobulin therapy administered 12 times was conducted for the PWCD after the IVIG therapy, not for the allergic diseases as described at the end of this section.

### 2.2 Histobulin therapy in Pfeiffer‐Weber‐Christian Disease (PWCD): the 2nd round of Histobulin therapy for PWCD

During the 1st cycle of Histobulin therapy, there was an improvement in her clinical symptoms and signs such as a reduction in the size of her breast mass and excruciating pain. Immediately, she canceled the plan for chemotherapy for lymphoma and her diagnosis of the breast mass began to be revised. Considering her overall clinical progress and pathologic findings, her final diagnosis for the breast mass was PWCD. Her PWCD breast mass‐related disease was improved with the 1st cycle of Histobulin therapy. However, in spite of the improvement of the pre‐existing lesion by Histobulin therapy, a new small mass was then developed. During the first cycle, the relationship between the Histobulin therapy and the improvement of PWCD could not be considered based on general medical knowledge and a review of the published literature.

The effects of Histobulin were suspected as being due to the effects of the small quantity of immunoglobulin in Histobulin,^4^ and IVIG therapy was conducted as described in a previous report.^9^ Her subjective symptoms were improved immediately and dramatically by IVIG therapy. She received IVIG (Liv‐Gamma SN inj, SK plasma, Korea) for 3 months according to the schedule of 1 g/kg 4 times every week, 1 g/kg twice every other week, and 400 mg/kg twice every other week. Her symptoms and signs, including the mass size and excruciating pain, were considerably improved. However, some small masses caused some discomfort, which was not painful, but they were not resolved and remained unchanged.

At this point, she appealed to the differences in her clinical and physical changes between the Histobulin therapy and the IVIG therapy without detailed descriptions. She just described that she was improved but new lesions were developing after stopping the Histobulin therapy and that while she showed immediate and dramatic improvement after IVIG therapy, some lesions with discomfort remained.

Considering the clinical improvement after the 1st Histobulin therapy, Histobulin therapy was reintroduced every week for a total of 12 weeks. Surprisingly, at the end of the 2nd cycle of Histobulin therapy, the remaining masses had disappeared and all clinical symptoms and signs, including the discomfort of the lesions, had subsided, resulting in complete remission of her PWCD.

After the 2nd cycle of Histobulin therapy for the PWCD, her atopic dermatitis was remitted and her SCORad index was 0 points (Table 1). All symptoms and signs of allergic rhinitis had disappeared. Most of all, she showed no allergic reactions after eating crab, shrimp, and mackerel. In the final follow‐up test after finishing the 2nd cycle of Histobulin therapy for PWCD, her complete blood counts with differential counts and serum eosinophilic cationic protein were within the normal ranges but her serum total IgE level was elevated to 2277 IU/mL. The specific IgE and skin prick test results for allergens were as described in Table 1. Especially, her specific IgE for crab was decreased and that for mackerel was converted to negative. Concerning upper respiratory infections, she suffered from upper respiratory infections including simultaneous rhinitis around 20 days per month for the last 3 years before the 1st cycle of Histobulin therapy and she did not suffer from any upper respiratory infections during and 3 months after the IVIG and Histobulin therapy.

## 3 DISCUSSION

### 3.1 Histobulin therapy in Pfeiffer‐Weber‐Christian Disease

Until now, there has been no uniformly effective therapy for PWCD. Corticosteroids ^10^ and immunosuppressive agents such as cyclosporine A ^11^ have been mainly used for its treatment. However, in this case, she was resistant to steroids and cyclosporine A. In this case, IVIG therapy was very effective and its effects were immediate and dramatic. However, IVIG could not resolve the disease completely.

The action mechanisms and relevant effects of Histobulin and IVIG seem to be different. In the first cycle, the Histobulin effects on PWCD were not considered because new lesions with systemic symptoms and signs, including excruciating pain, had developed. Histobulin contains just 12.5 mg of immunoglobulin. The improving effects were suspected to be due to the small dose of immunoglobulin in Histobulin, and high dose IVIG was considered. The IVIG was effective but some lesions with weak symptoms and signs persisted. According to the patient's obscure description concerning the difference of her clinical responses between Histobulin therapy and IVIG therapy, Histobulin therapy was reintroduced and a remission of PWCD was achieved. The effects of Histobulin were not the simply due to the effects of the small quantity of immunoglobulin in Histobulin. So, complete induction of remission in PWCD needed both Histobulin therapy and IVIG therapy.

Before this case was reported, there was only one previous report of an IVIG trial, but it used a combination with steroids.^12^ However, the effects of sole IVIG have not been described. IVIG was effective against PWCD as reported previously. However, IVIG therapy was not sufficient. Also, it is not clear whether the pretreatment with Histobulin for allergy treatment affected the IVIG therapy or not.

IVIG has anti‐inflammatory effects and has also been used against autoimmune diseases.^13^ There are no reports that Histobulin has anti‐inflammatory effects. So, considering the effects of Histobulin against PWCD, Histobulin seems to have anti‐autoimmune effects as well as anti‐inflammatory effects. Accordingly, PWCD seems to be both an immunologic disease ^14^, ^15^ and possibly an autoimmune disease.^16^ The clinical results of this case provide very important clues that Histobulin has the potential to be used for the treatment of autoimmune diseases. Moreover, combination therapy with Histobulin and IVIG against autoimmune disease needs to be further investigated and applied clinically.

**Figure 1:**
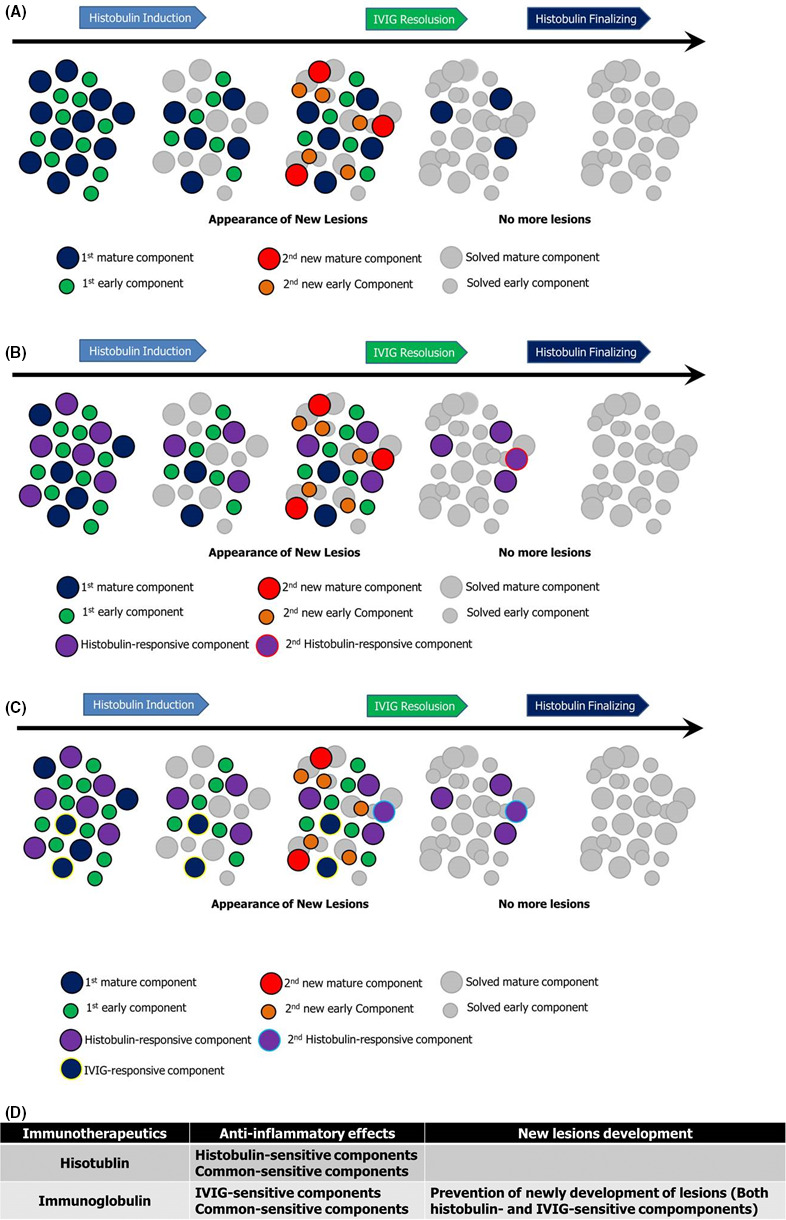
Immunotherapy for PWCD with allergic diseases was performed with Histobulin and IVIG. By the end of the 1st cycle of Histobulin therapy (Histobulin induction), her masses were reduced in size but new lesions developed. IVIG therapy (IVIG resolution) showed immediate and dramatic improvements but some small persistently unresolved and unchanged masses were left. By the end of the 2nd cycle of Histobulin therapy (Histobulin finalizing), the remaining unresolved masses had disappeared. The mechanisms were suspected sequentially as described. A, Prevention of new development of lesions by IVIG but not by Histobulin. The lesions were improved with the 1st cycle of Histobulin therapy. However, new lesions appeared, which were resistant to Histobulin. Here, it is concluded that IVIG also improved the lesions and prevented the development of new lesions. B, Presence of Histobulin‐responsive components in the lesions. Histobulin‐responsive components were present. Initially, the Histobulin‐responsive components were removed by the 1st cycle of Histobulin therapy. However, the new lesions, including Histobulin‐responsive components, developed. By IVIG therapy, the lesions were improved, leaving the Histobulin‐responsive components that were removed by the 2nd cycle of Histobulin therapy. C, Commonly responsive components to Histobulin and IVIG and IVIG‐responsive components were present. Both general symptoms and signs were dramatically improved, and the mass sizes were remarkably reduced by Histobulin and IVIG therapy, although the progress showed some differences. So there seem to be commonly responsive components. However, in the 1st cycle of Histobulin therapy, new lesions developed and these lesions subsided during IVIG therapy. So, IVIG‐responsive components are also present. D, Suspected immunopathogenetic action mechanisms of IVIG and Histobulin in PWCD

Without any intention, the treatment of PWCD in this case was conducted as a therapeutic protocol: induction by Histobulin therapy, IVIG therapy, and finalizing by Histobulin (Figure 1A). Clinically, new lesions developed during the 1st Histobulin therapy, but not during or after the IVIG therapy. Histobulin showed therapeutic effects in PWCD but could not prevent the new development of lesions. On the contrary, some small lesions remained unchanged without the development of new lesions after IVIG therapy. The remaining lesions were resolved by the 2nd cycle of Histobulin therapy (Figure 1A). IVIG seems to have both anti‐inflammatory effects on the lesions and to prevent new lesions from forming. This is relevant to the effects of IVIG against anti‐inflammatory and anti‐autoimmune disease if PWCD is an autoimmune disease. However, some lesions remained persistently unchanged in spite of extensive IVIG therapy and these unresolved lesions were cleared by the 2nd cycle of Histobulin therapy. It seems to be that there may be three kinds of components in PWCD, as one component is solved by IVIG, the other component is solved by Histobulin, and another component is solved in common by both IVIG and Histobulin (Figure 1B,C). But only IVIG prevented the development of new lesions. Both mechanisms may work together. It seems to be clear that both IVIG and Histobulin are necessary for the remission of PWCD (Figure 1D), and further investigation into the pathogenic mechanisms may be necessary.

### 3.2 Histobulin therapy in allergic disease

Multiple allergic diseases improved simultaneously in response to Histobulin therapy in this case report. Histobulin has been used to treat allergic rhinitis and atopic dermatitis without any clear action mechanisms.^4^ Recently, Histobulin was reported to useful in treating atopic dermatitis.^5^ In this case, Histobulin improved the patient's severe atopic dermatitis considerably to remission and the Histobulin effectiveness was reconfirmed. Also, her allergic rhinitis was improved to symptom‐free status. Moreover, her multiple food allergies were also resolved and the patient did not show any allergic symptoms and signs after intake of her previously allergenic foods. In the laboratory tests, the specific IgE for crab was decreased and that for mackerel was converted to negative.

In this case, not only was her total IgE decreased by Histobulin therapy, but also her specific IgE for allergens was decreased or converted to negative by the Histobulin therapy (Table 1). Histobulin seems to have non–allergen‐specific effects by lowering the specific IgE for allergens regardless of which allergens, which seems to be a mechanism for non–allergen‐specific desensitization effects as compared to allergen‐specific desensitization.^17^ Poly‐desensitization effects have been reported after IFN‐γ therapy,^18^ and Histobulin also seems to have poly‐desensitization effects.

**Figure 2:**
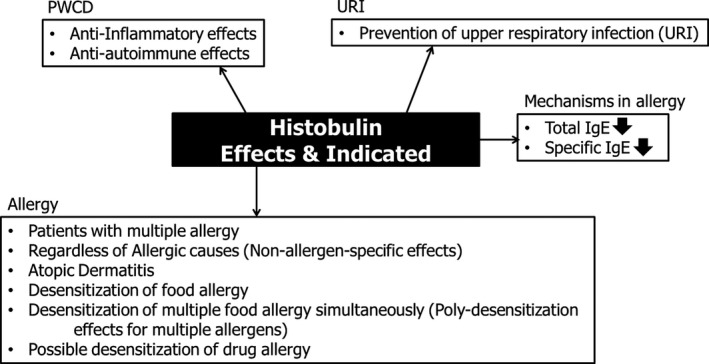
Clinical implications of the results of this case. The effects, the mechanisms, and the possible extended clinical indications of Histobulin in PWCD and allergic diseases

These results imply several points about the effects of Histobulin in the treatment of allergic diseases (Figure 2). (a) Histobulin is appropriate for a patient who has multiple allergic diseases. (b) Histobulin effects seem to be effective against allergic disease regardless of the causes (non–allergen‐specific effects). (c) Histobulin is effective against atopic dermatitis. (d) Allergies against multiple foods were resolved simultaneously. This is a very important result because Histobulin seems to solve allergies against specific foods. (e) Histobulin has poly‐desensitization effects. The only way to solve food and drug allergies is desensitization,^17^ except for growing out of them or a natural resolution. Drug allergy is a severe issue that needs to be solved. Histobulin may have the potential for desensitization of drug allergies, and further clinical investigations and trials may be needed.

### 3.3 Reduced frequency of upper respiratory infections by Histobulin therapy

In a previous report, a considerable reduction in the frequency of upper respiratory infections was reported after Histobulin therapy.^6^ This was found during Histobulin therapy for AD. In this case, the same result was obtained. These clinical results mean that Histobulin seems to have preventive effects against upper respiratory infections. There have been no reports concerning any preventive medications for upper respiratory infections.

This also suggests the possibility to use Histobulin as a preventive therapeutic against pandemic viral infections such as SARS‐CoV‐1 and ‐2, at least for allergic patients or subjects who have allergic tendencies because allergic conditions increase the risk of upper respiratory infections^19^. In this uncontrolled situation without a specific vaccine or therapeutic, the possibility of Histobulin as a temporary preventive therapeutic needs to be investigated.

These clinical results were obtained in a patient with PWCD with multiple allergic diseases. Currently, there are no reports concerning the reactions and interactions between IVIG therapy and Histobulin therapy, but this should be considered in the future.

## 4 CONCLUSIONS

Histobulin was a complementary but essential therapeutic for PWCD and Histobulin is also a potential therapeutic for the treatment of autoimmune diseases. Also, combination therapy with Histobulin and IVIG for autoimmune diseases needs to be further investigated and applied clinically.

Histobulin is appropriate for a patient who has multiple allergic diseases. The effects of Histobulin seem to be effective against allergic disease regardless of the cause. Histobulin is effective against atopic dermatitis, consistent with a previous report. Histobulin desensitized multiple food allergens simultaneously and also had an effect on allergen‐specific allergies by decreasing allergen‐specific IgE and total IgE.

Histobulin seems to have preventive effects against upper respiratory infections in this case and in previous cases. Histobulin needs to be investigated as a preventive therapeutic against pandemic viral infections such as SARS‐CoVID‐1 and −2, at least for allergic patients or subjects who have allergic tendencies.

## CONFLICT OF INTEREST

The author declares that there is no conflict of interest regarding the publication of this manuscript.

## AUTHOR CONTRIBUTIONS

Geunwoong Noh: is the only author of this manuscript.

## Data Availability

None.

## References

[1] Pfeifer V . Über einen Fall von herdweiser Atrophie des subkutanen Fettgewebes. Deutsches Archiv für klinische Medizin. 1892;438‐449.

[2] Weber EP . A case or relapsing nonsuppurative nodular panniculitis. Brit J Derm. 1925;37:301‐311.

[3] Christian HA . Relapsing febrile nodular nonsuppurative panniculitis. Arch Intern Med. 1928;41:338‐351.

[4] Kim JH , Shin IS , Lee YK , Oh HJ , Ban SJ . Improved HPLC Method Using 2,3‐naphthalenedicarboxaldehyde as Fluorescent Labeling Agent for Quantification of Histamine in Human Immunoglobulin Preparations. Osong public Health Res Perspect. 2011;2:127‐134. 24159462 10.1016/j.phrp.2011.07.003 PMC3766917

[5] Gushcin IS , Luss LV , Il'ina NI , et al. Therapeutic effectiveness of histaglobin preparations in patients with allergic rhinitis and chronic urticaria. Ter Arkh. 1999;71:57‐62. 10234769

[6] Noh G . Immunotherapy using Histobulin in atopic dermatitis. Clin Case Rep. 2020. In press. 10.1002/ccr3.3472 PMC7813077 33489144

[7] Hanifin JM , Rajka G . Diagnostic features of atopic dermatitis. Acta Derm Venereol. 1980;92:44‐47.

[8] Sprikkelman AB , Tupker RA , Burgerhof H , et al. Severity scoring of atopic dermatitis: A comparison of three scoring systems. Allergy. 1997;52:944‐949. 9298180 10.1111/j.1398-9995.1997.tb01255.x

[9] Noh G , Han CW . Intravenous Immune Globulin (IVIG) Therapy After Unsuccessful Treatment with Corticosteroid and Cyclosporine A in Pfeifer‐Weber‐Christian Disease: A Case Report. Am J Case Rep. 2021. 10.12659/AJCR.929519 PMC7791466 33390586

[10] Wang Y , Zhao J , Ji LL , Zhang S , Zhang Z . Weber‐Christian disease presenting with lung nodules dramatically improved with corticosteroid therapy: one case report and literature review. Int J Rheum Dis. 2018;21:573‐578. 26224252 10.1111/1756-185X.12620

[11] Pongratz G , Ehrenstein B , Hartung W , Scholmerich J , Fleck M . A patient with Pfeifer‐Weber‐Christian disease–successful therapy with cyclosporin A: case report. BMC Musculoskelet Disord. 2010;11:18. 20105325 10.1186/1471-2474-11-18 PMC2828421

[12] Wu F , Zou CC . Childhood Weber‐Christian Disease: Clinical Investigation and Virus Detection. Acta Paediatr. 2007;96:1665‐1669. 17888054 10.1111/j.1651-2227.2007.00498.x

[13] Galeotti C , Kaveri SV , Bayry J , et al. IVIG‐mediated effector functions in autoimmune and inflammatory diseases. Int Immunol. 2017;29:491‐498. 28666326 10.1093/intimm/dxx039

[14] Kumar R , Dayal D , Kumar S , et al. Weber‐Christian Panniculitis: Is it a Disorder of Immune System? Indian J Pediatr. 2016;83:1033‐1034. 26944431 10.1007/s12098-016-2067-5

[15] Iwatsuki K , Tagami H , Yamada M . Weber‐Christian panniculitis with immunological abnormalities. Dermatologica. 1982;164:181‐188. 7044852 10.1159/000250088

[16] Brázdilová K , Čierny D , Hrubišková K , Plank L , Killinger Z , Payer J . Weber‐Christian disease: a case report. Vnitr Lek. 2018;64:961‐965. 30590944

[17] Noh G , Lee SS . A pilot study of interferon‐gamma‐induced specific oral tolerance induction (ISOTI) for immunoglobulin E‐ mediated anaphylactic food allergy. J Interferon Cytokine Res. 2009;29(10):667‐675. 19642905 10.1089/jir.2009.0001

[18] Lee JH , Noh G . Polydesensitisation with reducing elevated serum total IgE by IFN‐gamma therapy in atopic dermatitis: IFN‐gamma and polydesensitisation (PDS). Cytokine. 2013;64:395‐403. 23786862 10.1016/j.cyto.2013.05.011

[19] Edwards MR , Strong K , Cameron A , Walton RP , Jackson DJ , Johnston SL . Viral infections in allergy and immunology: How allergic inflammation influences viral infections and illness. J Allergy Clin Immunol. 2017;140:909‐920. 28987220 10.1016/j.jaci.2017.07.025 PMC7173222

